# Investigation of Novel RNA Elements in the 3′UTR of Tobacco Necrosis Virus-D

**DOI:** 10.3390/v12080856

**Published:** 2020-08-06

**Authors:** Laura R. Newburn, Baodong Wu, K. Andrew White

**Affiliations:** Department of Biology, York University, Toronto, ON M3J 1P3, Canada; lnewburn@yorku.ca (L.R.N.); baodongw@gmail.com (B.W.)

**Keywords:** plant virus, RNA virus, 3′UTR, recoding, readthrough, cap-independent translation, RNA structure, tombusviridae, betanecrovirus

## Abstract

RNA elements in the untranslated regions of plus-strand RNA viruses can control a variety of viral processes including translation, replication, packaging, and subgenomic mRNA production. The 3′ untranslated region (3′UTR) of Tobacco necrosis virus strain D (TNV-D; genus *Betanecrovirus*, family *Tombusviridae*) contains several well studied regulatory RNA elements. Here, we explore a previously unexamined region of the viral 3′UTR, the sequence located upstream of the 3′-cap independent translation enhancer (3′CITE). Our results indicate that (i) a long-range RNA–RNA interaction between an internal RNA element and the 3′UTR facilitates translational readthrough, and may also promote viral RNA synthesis; (ii) a conserved RNA hairpin, SLX, is required for efficient genome accumulation; and (iii) an adenine-rich region upstream of the 3′CITE is dispensable, but can modulate genome accumulation. These findings identified novel regulatory RNA elements in the 3′UTR of the TNV-D genome that are important for virus survival.

## 1. Introduction

Tobacco necrosis virus strain D (TNV-D), and other plus-strand RNA plant viruses contain RNA elements throughout their genomes that regulate different viral processes [1,2,3,4,5,6,7,8]. Although some regulatory elements can reside in coding regions, most are located in non-coding regions, where they do not have to maintain compatibility with viral open reading frames (ORFs). Consequently, many such RNA sequences and structures are present in the 5′ and 3′ untranslated regions (UTRs) of viral genomes.

**Figure 1:**
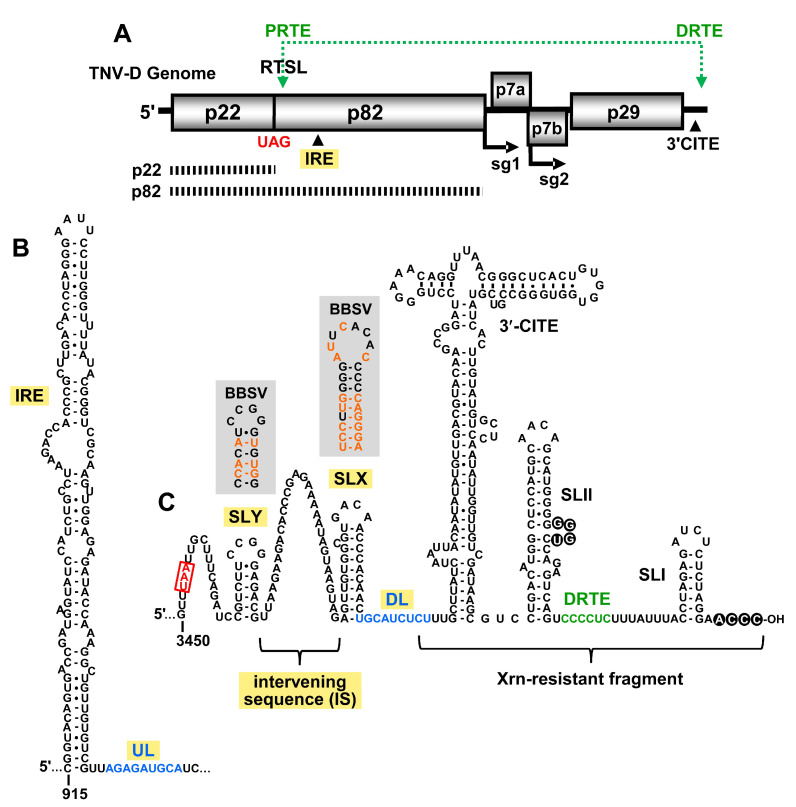
Tobacco necrosis virus strain D (TNV-D) genome and the predicted RNA secondary structures. (**A**) A cartoon representation of the TNV-D genome with the encoded proteins represented by grey boxes. The UAG stop codon for p22 is indicated in red. Subgenomic mRNA1 and 2 initiation sites (sg1 and sg2) are shown below the genome. The location of the 3′ cap independent translational enhancer (3′CITE) and the internal replication element (IRE) are also shown. The black dashed bars below the genome correspond to the p22 protein and the p82 readthrough product. The green double-headed arrow shows a long-range RNA–RNA interaction between the proximal readthrough element (PRTE; located within the readthrough stem–loop, RTSL) and the distal readthrough element (DRTE; in the 3′UTR) that is necessary for efficient readthrough. (**B**) Mfold-predicted RNA secondary structure of the TNV-D internal replication element (IRE), located within the coding region of p82. The 3′-adjacent upstream linker (UL) sequence is shown in blue. (**C**) Predicted RNA secondary structure of the 3′UTR in the TNV-D genome based on comparative structural analysis, compensatory mutational analysis, and structure probing data [2,5,6,7]. The UAA stop codon of the p29 capsid protein is boxed and highlighted in red, and the portion of the 3′UTR that accumulates as an Xrn-resistant fragment in infections is delineated. The 3′CITE and relevant stem–loop (SL) structures are labelled, as are the intervening sequence (IS), downstream linker (DL; blue) and DRTE (green). The grey boxes show the corresponding secondary structures of SLX and SLY for Beet black scorch virus (BBSV), with orange nucleotides representing the sequence changes when compared with TNV-D.

TNV-D is a plus-sense RNA plant virus in the family *Tombusviridae*, and is the type-member of the genus *Betanecrovirus,* which also includes Beet black scorch virus (BBSV) [9]. The genome of TNV-D is 3.8 kb in length and contains five open reading frames (ORFs) (Figure 1A) [10]. Proteins that are produced directly from the viral genome include the accessory RNA replication protein (p22), and the RNA-dependent RNA polymerase (RdRp; p82), produced via readthrough of the p22 stop codon [11,12]. Movement proteins p7a and p7b are translated from subgenomic (sg) mRNA1, and coat protein (CP; p29) is produced from sg mRNA2 [13].

Different regions of the 3′UTR of the TNV-D genome are involved in various viral processes, including translation initiation, translational readthrough, and RNA replication. The last 80 nt of the 3′UTR contains two stem–loop (SL) structures, SL-II and SL-I, that are separated by 15 nts. A base-pairing interaction between the 3′-terminal 4 nts and a bulge in SLII is important for efficient RNA synthesis, and likely protects the 3′-end of the genome from exonucleases (Figure 1C, black circles) [2,7]. Just 3′ to SLII resides the distal readthrough element (DRTE) (Figure 1C, green), a 6 nt long segment that activates readthrough of p82 by forming a long-range RNA–RNA interaction with its partner sequence, the proximal readthrough element (PRTE) (Figure 1A, top) [1]. The PRTE is a bulged sequence located within an extended SL structure, the readthrough SL (RTSL), that is positioned just downstream of the p22 UAG stop codon (Figure 1A). The PRTE–DRTE interaction is the only reported long-range RNA–RNA interaction known to occur in betanecroviruses [1].

The translation of TNV-D and its two sg mRNAs is mediated by a BTE-type 3′-cap independent translation enhancer (3′CITE) [3,6,7]. This 3′CITE is located in the central region of the 3′UTR (Figure 1C), and is responsible for recruiting the translational machinery to the 5′-uncapped and 3′-non-adenylated viral mRNAs. The mechanism by which the 3′-recruited translation factors gain access to the 5′-end of these viral messages is unknown [3]. During infections, an Xrn-resistant fragment accumulates, which corresponds to a 3′-proximal portion of the 3′UTR, including the 3′CITE (Figure 1C) [14]. The precise role of this 3′-terminal viral RNA fragment is unclear, but its presence appears to confer viral fitness [14].

In this study, we investigated the importance of RNA elements present in the previously unexplored 5′-portion of the 3′UTR of TNV-D. Our results provide evidence for a second long-range RNA–RNA interaction in the TNV-D genome that is important for viral RNA accumulation and translational readthrough. We also uncovered a conserved RNA hairpin, SLX, that is involved in supporting efficient viral replication. These findings further our understanding of RNA-based regulatory elements in betanecroviruses.

## 2. Materials and Methods

### 2.1. Plasmid Construction

Constructs for this study were derivatives of wild-type (wt) TNV-D cDNA in a pUC19 plasmid, provided by Robert Coutts [10], which was modified to include a SmaI cut site at the 3′-terminus of the viral cDNA. Overlapping and standard PCR-based mutagenesis was used to introduce modifications to the TNV-D clones, using the Q5 High-Fidelity DNA Polymerase kit (NEB). The inclusion of only desired alterations was confirmed through sequencing. Changes introduced to the TNV-D genome are shown in the accompanying figures.

### 2.2. Preparation of Viral RNAs

Uncapped in vitro RNA transcripts were synthesized from SmaI-linearized TNV-D plasmids using the T7-FlashScribeTranscription Kit (CELLSCRIPT), as previously described [15]. RNA transcript concentration and quality were verified through spectrophotometry and agarose gel electrophoresis, respectively.

### 2.3. RNA Secondary Structure Prediction

TNV-D RNA secondary structures were predicted at 37 °C using the default settings of *Mfold* version 3.6 [16,17].

### 2.4. In Vitro Translation Assay

As described previously, 0.5 pmol of uncapped in vitro-generated viral genomic RNA transcripts were incubated in a nuclease-treated wheat germ extract (Promega, Madison, WI, USA) in the presence of ^35^S-methionine for 1 h at 25 °C [1,2]. The protein products were separated by sodium dodecyl sulfate-polyacrylamide gel electrophoresis in a 12% resolving gel. Viral proteins produced were detected by radio-analytical scanning using a Typhoon imager (GE Healthcare, Chicago, IL, USA) and quantified using QuantityOne software (Bio-Rad, Hercules, CA, USA). All trials were completed at least three times. Averages with standard errors are shown in the accompanying figures.

### 2.5. Protoplast Infections

**Figure 2:**
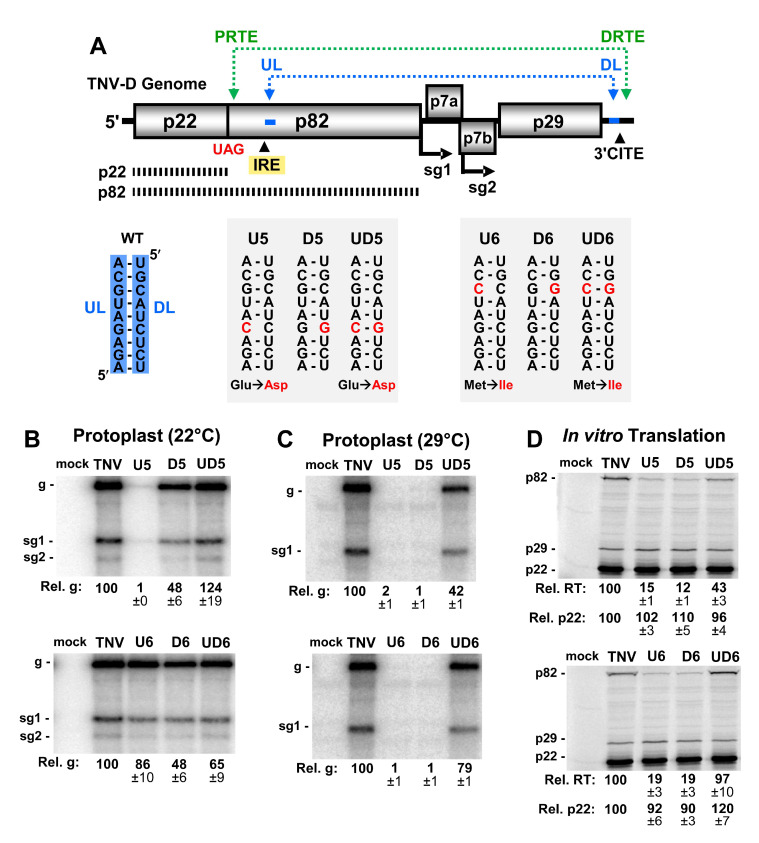
Analysis of the upstream linker (UL) and downstream linker (DL) interaction. (**A**) Cartoon representation of the TNV-D genome with the UL–DL interaction shown by a blue double-headed arrow. Wild-type nucleotide sequences of the UL–DL interaction are highlighted in blue, and nucleotide substitutions in mutants are shown in red, with corresponding amino acid changes below. (**B** and **C**) Northern blot analyses of TNV-D RNA accumulation in cucumber cotyledon protoplasts incubated for 22 h at either (**B**) 22 °C or (**C**) 29 °C. Above each lane is the name of the viral genome analyzed, with the positions of the viral genome (g) and subgenomic mRNAs (sg1 and sg2) denoted to the left of the blot. In this and subsequent experiments, the relative genome levels (Rel. g) of mutant genomes were determined by relative comparison to that of wt TNV-D (set at 100%) and are shown below each lane, as determined from three independent experiments (±standard error). (**D**) In vitro translation assay of TNV-D genomic RNA incubated in wheat germ extract. Viral constructs tested are noted above their respective lanes, and the identities of the protein products (p82, p29, and p22) are shown on the left. Relative readthrough (Rel. RT) was calculated as the ratio of p82/p22, with that for the wild-type genome set as 100%. Relative p22 levels (Rel. p22) were determined by direct comparison of wt (100%) versus mutant levels. The corresponding means (±standard errors) were calculated based on three independent assays.

**Figure 3:**
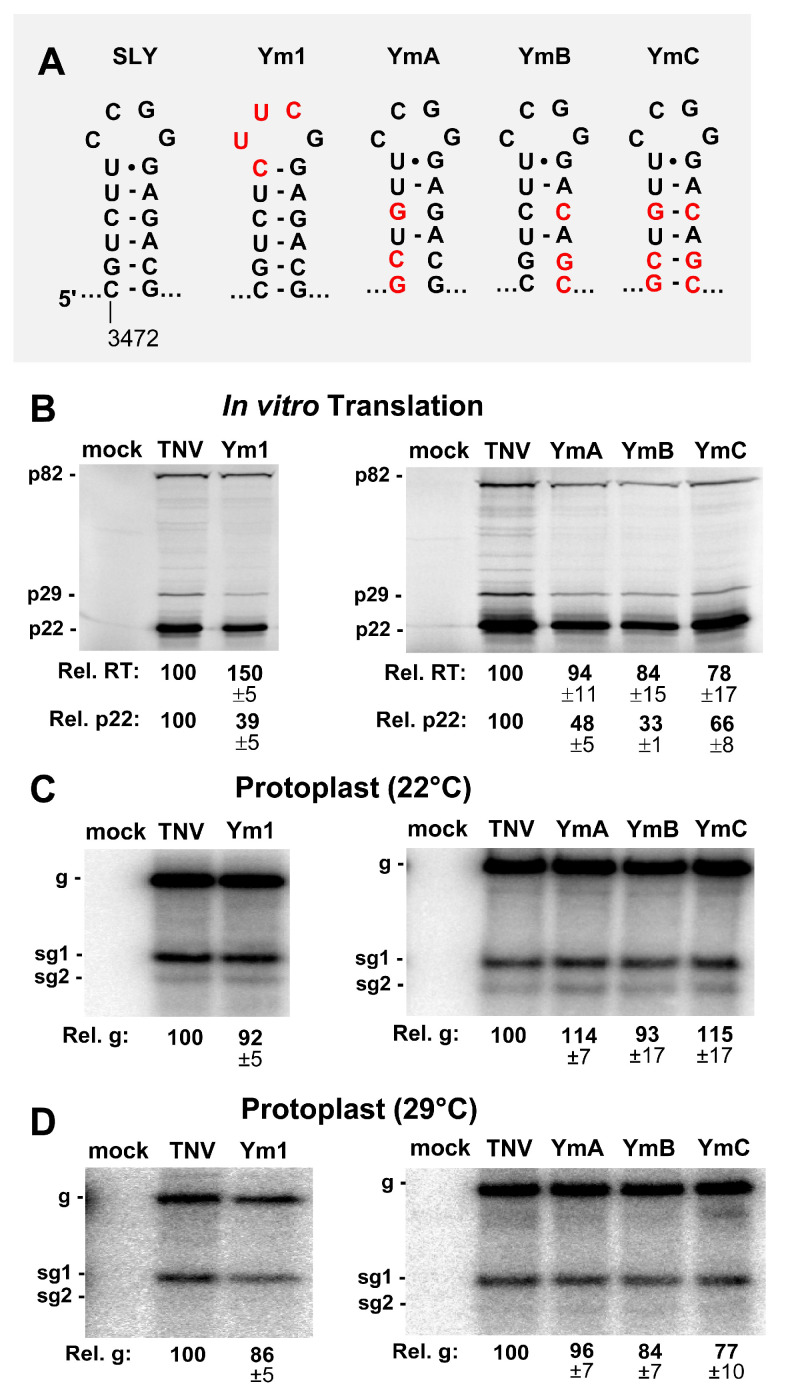
Mutational analysis of the SLY. (**A**) Wild-type and mutant SLYs, with substituted nucleotides in red. (**B**) In vitro translation analysis of the SLY mutants in panel A. (**C** and **D**) Northern blot analysis of the protoplast infections with SLY mutants at (**C**) 22 °C and (**D**) 29 °C.

**Figure 4:**
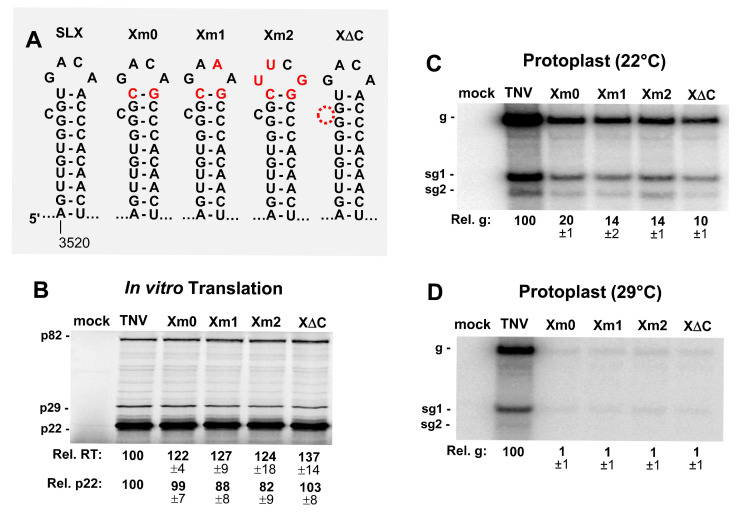
Mutational analysis of the loop and bulge of SLX. (**A**) Wild-type and mutant SLXs, with substituted nucleotides in red. (**B**) In vitro translation analysis of the SLX mutants. (**C** and **D**) Northern blot analysis of the protoplast infections with SLX mutants at (**C**) 22 °C and (**D**) 29 °C.

**Figure 5:**
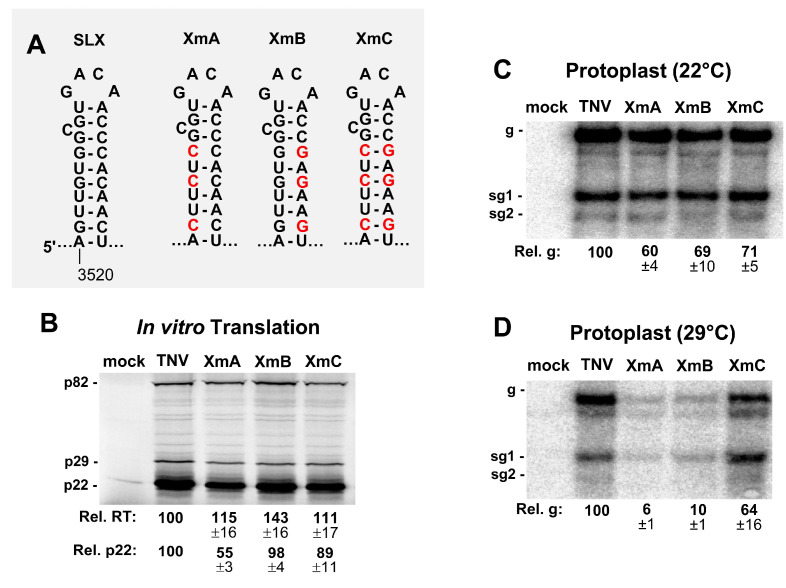
Mutational analysis of the stem of SLX. (**A**) Wild-type and mutant SLXs, with substituted nucleotides in red. (**B**) In vitro translation analysis of the SLX mutants. (**C** and **D**) Northern blot analysis of the protoplast infections with SLX mutants at (**C**) 22 °C and (**D**) 29 °C.

**Figure 6:**
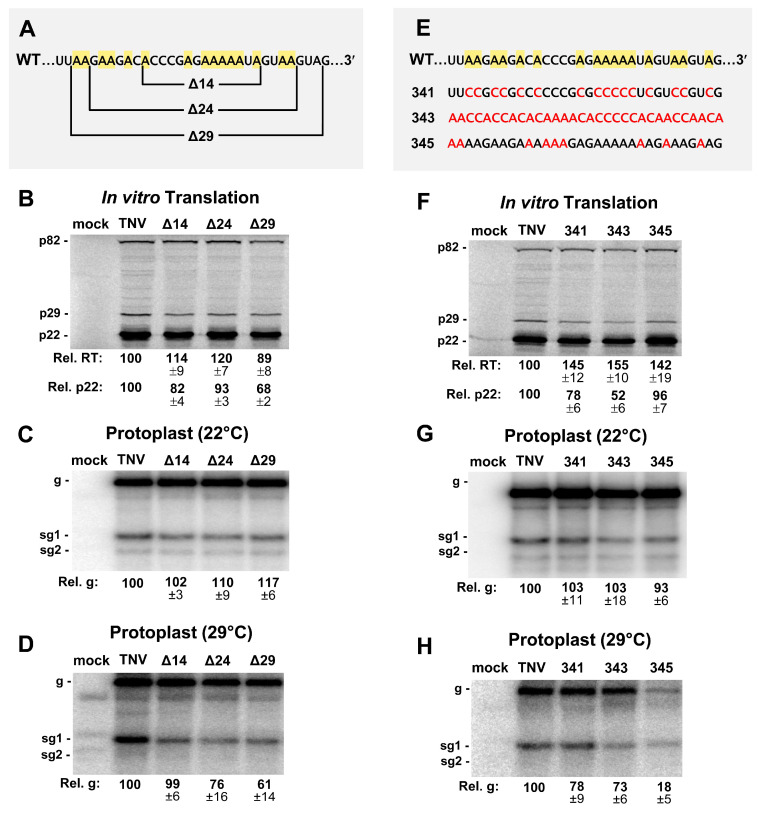
Mutational analysis of the intervening sequence (IS). (**A**) Deletions made in the 32 nucleotide long IS region (Δ14, Δ24, Δ29). Adenylate residues are highlighted in yellow in the wt sequence. (**B**) In vitro translation analysis of the IS mutants in panel A. (**C** and **D**) Northern blot analysis of protoplast infections with deletion mutants in panel A at (**C**) 22 °C and (**D**) 29 °C. (**E**) Substitutions in the IS are shown in red (341, 343, 345) and adenylate residues are highlighted in yellow in the wt sequence. (**F**) In vitro translation analysis of IS mutants in panel E. (**G** and **H**) Northern blot analysis of protoplast infections with deletion mutants in panel E at (**G**) 22 °C and (**H**) 29 °C.

Cucumber cotyledon protoplasts were prepared and transfected with uncapped in vitro-generated viral genomic RNA transcripts as described previously [18]. Briefly, 3 µg of viral genomic RNA was transfected into protoplasts using polyethylene glycol and incubated under constant light for 22 h at 22 or 29 °C, as indicated in each figure. Total nucleic acid extraction was performed as described previously [18], and separated in non-denaturing 2% agarose gels. The nucleic acids were electro-transferred from the agarose gel to a nylon membrane and viral RNAs were detected through Northern blotting using ^32^P-radiolabeled probes designed to hybridize to the 3′UTR of TNV-D. Since the constructs used in this study had altered 3′UTR sequences, different probes were used to avoid overlap with the positions of the modifications. For the upstream linker (UL)–downstream linker (DL) modifications (Figure 2), three oligonucleotide probes were used; pTN14 (complementary to nt 2821–2840), pTN8 (complementary to nt 3512–3531), and pTN10 (complementary to nt 3643–3663). For the SLX and SLY modifications (Figure 3, Figure 4 and Figure 5), two probes were used; pTN14 and pTN10. For all intervening sequence modifications (Figure 6), two probes were used; pTN14 and pL205 (complementary to nt 3568–3588). Viral RNA accumulation was monitored using a Typhoon imager (GE Healthcare) and quantified with QuantityOne software (Bio-Rad). All trials were completed three times, and averages with standard errors are shown in the accompanying figures.

## 3. Results

### 3.1. A Long-Range RNA–RNA Interaction Promotes Genome Accumulation and Translational Readthrough

Several members of the family *Tombusviridae*, including TNV-D, utilize an internal replication element (IRE) located in the readthrough portion of their RdRp ORFs (Figure 1A), which forms an extended RNA stem–loop structure (Figure 1B) [4]. In tombusviruses, the IRE (termed RII-SL) is bound by dimers of the auxiliary replication protein p33, which corresponds to p22 in TNV-D [19,20,21]. In Tomato bushy stunt virus (TBSV), the sequence 3′-adjacent to the base of the RII-SL structure, termed the upstream linker (UL), engages in a long-range base pairing interaction with a sequence in the genomic 3′UTR, called the downstream linker (DL) [22]. This interaction positions RII-SL proximal to another important replication structure, RIV, located at the 3′ end of the genome. The bipartite RII-SL/RIV structure forms an RNA platform on which the RNA replication complex assembles [22]. Accordingly, the UL–DL interaction is required for tombusvirus RNA synthesis and accumulation in infections. The UL–DL interaction also promotes efficient readthrough-based production of the RdRp [23].

Analysis of the sequence 3′-adjacent to the IRE in the TNV-D genome and sequences within the 3′UTR revealed a possible base-pairing interaction similar to the UL–DL interaction in tombusviruses. A 9 nt-long UL sequence immediately downstream of the IRE was found to be complementary to a DL segment positioned just upstream of the 3′CITE (Figure 1B,C respectively, blue nucleotides). To functionally assess the putative UL–DL interaction, the partner sequences were subjected to compensatory mutational analysis in which complementarity was disrupted and then restored. Two sets of mutants were generated, each of which targeted a different base pair (Figure 2A). Both of the substitutions introduced into the UL sequence in mutants U5 and U6 resulted in single amino acid changes in the RdRp (Figure 2A), however, neither residue is highly conserved in members of *Tombusviridae* [24]. Initially, viral replication was assessed in protoplast infections incubated at 22 °C for 22 hr (Figure 2B). While one set of mutations (U6, D6, and UD6) did not show a correlative trend (Figure 2B, lower panel), the other set (U5, D5, and UD5) exhibited a dependence on complementarity between the partner sequences (Figure 2B, upper panel). At a higher incubation temperature of 29 °C, where the requirement for base pairing stability is more stringent, both sets of mutants showed a clear requisite for the UL–DL interaction (Figure 2C).

When the same genomic mutants were tested in in vitro translation assays, the production of the viral auxiliary replication protein p22 was moderately affected, whereas the efficiency of readthrough-production of p82 correlated positively with complementarity (Figure 2D). These results indicate that the UL–DL interaction is functionally important for both viral genome accumulation in protoplasts, and translational readthrough in vitro.

### 3.2. SLY Affects Translation In Vitro but not Viral RNA Accumulation in Protoplasts

Two SL structures, SLX and SLY, separated by a 32 nt A-rich intervening sequence (IS), were predicted upstream of the DL sequence (Figure 1C). The relevance of these SLs in TNV-D was supported by corresponding SLs at equivalent positions in genus-member BBSV, which contained numerous co-varying base pairs within the stems (Figure 1C, shaded boxes). The SLY terminal loops in both viruses were identical, whereas those in SLX were unique (Figure 1C).

The function of SLY was examined by introducing modifications into its loop or stem regions. The terminal loop of SLY was replaced by an ultra-stable tetraloop, UNCG, in genome mutant Ym1 (Figure 3A). The in vitro translation of Ym1 yielded decreased levels of p22, to ~39% of wt, while readthrough efficiency was elevated by a third (Figure 3B, left panel). When the stem of SLY was disrupted in YmA and YmB, p22 production fell to ~33–48% of wt, and recovered partially (~66%) in the compensatory mutant YmC (Figure 3B, right panel), while all three mutants exhibited moderately decreased readthrough-based production of p82 (Figure 3B). When assessed in protoplast infections, all SLY mutants showed comparatively modest increases or decreases in genome levels (Figure 3C,D). Accordingly, the more pronounced negative effects of the SLY alterations on translation initiation in vitro were not correspondingly evident in protoplast infections.

### 3.3. SLX Is Important for Viral RNA Accumulation in Protoplast Infections

SLX is predicted to contain a 4 nt terminal loop and a single nucleotide C bulge. Initially, the closing base pair of the terminal loop was altered from a UA to a CG in mutant Xm0, to introduce an optimal context for both UNCG and GNRA-type ultra-stable tetraloops (Figure 4A). For Xm0, and when the loop substitutions were added in Xm1 and Xm2, moderate decreases in p22 translation were observed (Figure 4B). Similarly, the deletion of the C bulge in X**Δ**C did not notably influence translation levels, however, all of the mutations appeared to modestly enhance readthrough (Figure 4B). When these genome mutants were assessed in protoplast infections incubated at 22 °C, accumulation levels were considerably reduced in all cases, to ~10–20% of wt, and the inhibition was exacerbated when the incubation temperature was increased to 29 °C (Figure 4C,D). Accordingly, the loop and bulge in SLX are important for viral RNA accumulation.

To investigate the functional relevance of the stem of SLX, compensatory mutations were introduced into this region (Figure 5A). In vitro translation assays showed elevated levels of readthrough (Figure 5B), similar to the loop mutants (Figure 4B). The production of p22 was modestly affected, with the exception of XmA, which showed about a 50% reduction (Figure 5B). When these constructs were tested for genome accumulation in protoplast infections incubated at 22 °C, genome accumulation decreased for all mutants to ~60–71% of wt (Figure 5C). However, when the incubation temperature was raised to 29 °C, a clear defect in genome accumulation was evident for XmA and XmB containing destabilized stems (~6% and ~10% of wt, respectively), with the compensatory mutant XmC showing recovery to ~64% (Figure 5C,D). Collectively, the results implicate all parts of SLX as being important for viral genome accumulation, with the upper region containing the loop and bulge being most functionally relevant.

### 3.4. The Intervening Sequence (IS) between SLX and SLY Is not Vital for Viral Translation or Infection

The intervening sequence (IS) between SLX and SLY is an A-rich segment with 16 of the 32 nucleotides being adenylates (Figure 1C). In Red clover necrotic mosaic virus (RCNMV) RNA1, an A-rich sequence is bound by poly-A binding protein (PABP) and, along with its 3′CITE, is critically important for facilitating translation of RCNMV RNA1 [25]. TNV-D has the same class of 3′CITE as RCNMV RNA1, termed BTE [6], and thus may also have a requirement for PABP recruitment. To test if the A-rich IS in TNV-D was important for viral function, sequential deletions of 14, 24, and 29 nucleotides were created in this segment (Figure 6A). When tested for in vitro translation, the effects were generally moderate (Figure 6B). Protoplast infections incubated at 22 °C yielded near wt levels of genome accumulation (Figure 6C), while at 29 °C, defects in genome accumulation, to ~76% and ~61% of wt, were observed when 24 or 29 nucleotides were deleted, respectively (Figure 6D).

To assess the importance of the IS independent of its length, substitutions were made that decreased (341) or increased (345) adenylate content, or shuffled (343) the wt sequence (Figure 6E). All changes resulted in increased translational readthrough by ~40–50%, and varying negative effects on translation initiation (~52–96% of wt) (Figure 6F). In protoplast infections at 22 °C, viral RNA accumulation was near wt (Figure 6G), however, at 29 °C, the effect was modestly negative for 341 and 343 (~78% and ~73%, respectively), but notably inhibitory for the A-enriched 345 (~18%) (Figure 6H). Overall, with the exception of mutant 345, the IS appears to moderately influence both translation in vitro and viral RNA accumulation in protoplast infections.

## 4. Discussion

The 3′UTRs of plus-strand RNA viruses have long been known to harbor regulatory RNA elements important for controlling viral processes [2,26]. Our analysis of unexplored regions within the 3′UTR of the TNV-D genome revealed novel elements important for viral function. These RNA elements and their possible roles in viral translation and replication are discussed below.

Our results have identified a UL–DL interaction in the TNV-D genome that unites its IRE with the 3′UTR (Figure 2). In tombusviruses, the corresponding UL–DL interaction forms an IRE-3′UTR RNA platform that mediates RNA replicase complex assembly [21,22]. Aureusviruses also have a similar requirement for an equivalent UL–DL interaction [27], which is not surprising, as they encode orthologous viral proteins in similar positions as tombusviruses, and are the genera most closely related to tombusviruses [28]. In contrast, betanecroviruses, like TNV-D, have genomes that are chimeric in origin, having accessory replication protein (p22) and RdRp (p82) related to counterparts in tombusviruses, and movement and capsid proteins orthologous to those in carmoviruses [9]. However, even with these differences, TNV-D retained the requirement for a UL–DL interaction. This suggests that although TNV-D encodes distinct carmovirus-like proteins in the 3′-half of its genome, its tombusvirus-like RNA replication proteins dictate the requirement of an IRE–3′UTR interaction which, as shown for tombusviruses [21,22], may facilitate RNA replicase complex assembly for TNV-D. Whether other tombusvirids with IREs [4] also employ long-range RNA-based communication with their 3′UTRs remains to be explored.

Readthrough in TNV-D relies on an essential PRTE–DRTE interaction (Figure 2A) [1]. However, its UL–DL interaction was also found to be necessary for optimal readthrough production of the RdRp in in vitro translation assays (Figure 2D), as was observed for tombusviruses [23]. In tombusviruses, the readthrough-promoting role of the UL–DL was also shown to be required during protoplast infections, as demonstrated using a defective interfering (DI) RNA reporter assay [23]. Corresponding in vivo confirmation was not possible with TNV-D, because no naturally-occurring or artificially-engineered DI RNAs exist for this virus. Thus, only in vitro data support a role for TNV-D’s UL–DL interaction in readthrough. If this interaction does indeed enhance readthrough during infections, it would likely do so by promoting and/or stabilizing the PRTE–DRTE interaction, due to the proximity of the two interactions (Figure 2A).

Tombusvirus genomes do not contain any functionally important secondary structures in the region immediately upstream from their DL sequences. In contrast, TNV-D is predicted to contain two conserved hairpins in this location. SLY is the first predicted RNA secondary structure downstream of the p29 stop codon in the TNV-D genome (Figure 1C). Modifications in SLY that altered its loop or disrupted its stem primarily led to reduced translation initiation in in vitro assays (Figure 3B). However, protoplast infections indicated relatively minor inhibitory effects on viral RNA accumulation, suggesting a negligible role for SLY in vivo (Figure 3C,D). Nonetheless, the conservation of SLY in TNV-D and BBSV (Figure 1C) implies that it likely does confer a selective advantage to these viruses, however, this benefit may not be discernable under our experimental conditions.

SLX is also conserved in both betanecroviruses (Figure 1C). Modifications in its loop, bulge, or stem regions increased readthrough variably in translation assays, along with mostly minor negative effects on translation initiation (Figure 5B and Figure 6B). Unlike for SLY, mutants of SLX exhibited readily observable phenotypes when assessed in protoplast infections (Figure 5C,D and Figure 6C,D). The bulge and loop mutations were highly inhibitory to viral RNA accumulation, but stem stability also contributed, albeit less notably. These results implicate the upper region of SLX as the key feature conferring function, with the stem likely acting in a supporting role to optimally present the upper structural features. Accordingly, to confer its function, the loop/bulge region of SLX likely interacts with either a viral or host protein or another region of the viral RNA. A search for complementary sequences to the loop/bulge region in the TNV-D genome did not yield any compelling candidates, thus, the latter possibility seems less likely. Therefore, the more probable role for SLX is in interacting with a protein factor that acts to facilitate viral RNA accumulation.

Some tombusvirids, in addition to their 3′CITE, require an interaction with PABP to enhance translation [25]. In the case of RCNMV RNA-1, an adenine-rich sequence located 5′-proximal to its 3′CITE binds to PABP and assists in the recruitment of translation initiation factors [25]. The similar positioning of the A-rich IS upstream of the 3′CITE in TNV-D suggested that it could function in a similar capacity. Variable effects on both translation initiation (~52–96%) and readthrough (~89–155%) were observed when sections of the IS were deleted or replaced (Figure 6B,F). However, major decreases in protein synthesis were expected if it played a critical role in translation initiation. In protoplast infections, moderate viral RNA inhibition was observed for most mutants (73–99%), but only at 29 °C (Figure 6C,D,G,H). The most adverse effects were seen for the largest deletion (Δ29, 61%) and the A-enriched mutant (345, 18%) (Figure 6D,H). The former result suggests that the IS may be important for the optimal spacing of other RNA elements, while the latter observation indicates that certain sequences and/or nucleotide content can be very detrimental to genome accumulation during infections.

## 5. Conclusions

This study provides an initial assessment of the functional relevance of RNA elements in the upstream third of TNV-D’s 3′UTR. The results revealed the presence of RNA elements that were either similar to other tombusvirids (UL–DL interaction) or unique to the betanecrovirus genus (SLX and SLY). Among the four RNA elements investigated, SLX and the DL (along with its partner, the UL) were most functionally relevant. The UL–DL interaction promotes readthrough, and may facilitate RNA replicase complex assembly (as demonstrated in tombusviruses [21,22]), while the unknown essential role of SLX in mediating viral RNA accumulation will be the focus of future studies.
